# Is There a Need for Viral Load Testing to Assess Treatment Failure in HIV-Infected Patients Who Are about to Change to Tenofovir-Based First-Line Antiretroviral Therapy? Programmatic Findings from Myanmar

**DOI:** 10.1371/journal.pone.0160616

**Published:** 2016-08-09

**Authors:** Nay Thiha, Palanivel Chinnakali, Anthony D. Harries, Myint Shwe, Thanumalaya Perumal Balathandan, Sai Thein Than Tun, Mrinalini Das, Htay Htay Tin, Yi Yi, François Xavier Babin, Thi Thi Lwin, Philippe Albert Clevenbergh

**Affiliations:** 1 International Union Against Tuberculosis and Lung Disease, Mandalay, Myanmar; 2 Jawaharlal Institute of Postgraduate Medical Education and Research (JIPMER), Puducherry, India; 3 International Union Against Tuberculosis and Lung Disease, Paris, France; 4 London School of Hygiene and Tropical Medicine, London, United Kingdom; 5 National AIDS program, Department of Health, Nay Pyi Taw, Myanmar; 6 Burnett Institute, Yangon, Myanmar; 7 Medécins sans Frontières, Doctors without borders-OCB, Mumbai, India; 8 National Health Laboratory, Department of Health, Yangon, Myanmar; 9 Public Health Laboratory, Department of Health, Mandalay, Myanmar; 10 FondationMereiux, Lyon, France; 11 Hopital Lariboisière Groupe Hospitalier Saint-Louis Lariboisière, Paris, France; University of Maryland School of Medicine, UNITED STATES

## Abstract

**Background:**

WHO recommends that stavudine is phased out of antiretroviral therapy (ART) programmes and replaced with tenofovir (TDF) for first-line treatment. In this context, the Integrated HIV Care Program, Myanmar, evaluated patients for ART failure using HIV RNA viral load (VL) before making the change. We aimed to determine prevalence and determinants of ART failure in those on first-line treatment.

**Methods:**

Patients retained on stavudine-based or zidovudine-based ART for >12 months with no clinical/immunological evidence of failure were offered VL testing from August 2012. Plasma samples were tested using real time PCR. Those with detectable VL>250 copies/ml on the first test were provided with adherence counseling and three months later a second test was performed with >1000 copies/ml indicating ART failure. We calculated the prevalence of ART failure and adjusted relative risks (aRR) to identify associated factors using log binomial regression.

**Results:**

Of 4934 patients tested, 4324 (87%) had an undetectable VL at the first test while 610 patients had a VL>250 copies/ml. Of these, 502 had a second VL test, of whom 321 had undetectable VL and 181 had >1000 copies/ml signifying ART failure. There were 108 who failed to have the second test. Altogether, there were 94% with an undetectable VL, 4% with ART failure and 2% who did not follow the VL testing algorithm. Risk factors for ART failure were age 15–24 years (aRR 2.4, 95% CI: 1.5–3.8) compared to 25–44 years and previous ART in the private sector (aRR 1.6, 95% CI: 1.2–2.2) compared to the public sector.

**Conclusions:**

This strategy of evaluating patients on first-line ART before changing to TDF was feasible and identified a small proportion with ART failure, and could be considered by HIV/AIDS programs in Myanmar and other countries.

## Introduction

The scale up of antiretroviral therapy (ART) in low- and middle-income countries (LMIC) in the last decade has been a remarkable public health success. By 2013, an estimated 11.7 million people living with HIV (PLHIV) were receiving ART, representing 36% coverage of the 32.6 million PLHIV in these countries [1]. Frequent guidance from the World Health Organization (WHO) in the form of international guidelines has underpinned the public health approach to ART scale up with an emphasis on standardized regimens for use in adults and children and standardized monitoring of therapy.

Both the 2010 and 2013 WHO Guidelines on the use of antiretroviral drugs for treating and preventing HIV infection have emphasized a) the phasing out of stavudine (d4T) and replacement with tenofovir (TDF) for first-line regimens and b) monitoring the response to ART by viral load [2,3]. Monitoring in the early years of ART scale up was done through clinical assessment and/or measurement of CD4-cell count, but sensitivity and specificity of this approach is low leading either to inappropriate switching to second line treatment or continuation on a failing first-line regimen [4]. HIV RNA viral load is the preferred option to diagnose and confirm ART failure and is a strong recommendation from WHO, [3] but this has yet to be implemented at scale because of expense and the need for sophisticated laboratory infrastructure.

Myanmar has a concentrated HIV epidemic with HIV transmission primarily occurring in high risk sexual contacts between sex workers and their clients, men who have sex with men and injecting drug users as well as their partners. ART has been gradually scaled up in the country and by the end of 2013, 67,643 patients were receiving therapy [5]. ART is provided through clinics run by government and clinics run by non-governmental organizations, one of which is the International Union Against Tuberculosis and Lung Disease (The Union). The Union’s “The Integrated HIV Care Program” started in 2005, and since that time PLHIV have been started and maintained on d4T-based and zidovudine (AZT)-based first line ART and monitored through clinical assessment and CD4 count testing. Viral load testing has not been routinely available.

In line with the recommendation from Myanmar National guidelines, it was decided in 2012 to change all PLHIV retained in care on first-line ART to a TDF-based regimen [6]. In many countries and programs, this change is just made without evaluating whether patients have failed their first-line regimen. However, in The Integrated HIV Care Program, Myanmar, it was decided that patients should be first evaluated for ART failure using HIV RNA viral load. The justification for this approach was a) to take stock of the prevalence of ART failure seven to eight years after the program had first started and b) to ensure that patients were placed on correct therapy–either TDF-3TC-EFV as first-line treatment or TDF-3TC-lopinavir/ritonavir (LPV/r) as second-line treatment.

The aim therefore of this study was to determine the prevalence and determinants of ART failure in those on first-line treatment for more than 12 months. Specific objectives in PLHIV who were retained in care on d4T-based or AZT-based ART regimens for 12 months or longer with no clinical or immunological evidence of failure were to: i) describe baseline demographic, clinical and immunological characteristics, ii) summarize the management and results of using a viral load screening algorithm to determine the presence of ART failure, and iii) evaluate risk factors associated with ART failure.

## Materials and Methods

### Ethics

Permission for the study was granted by the National AIDS Program, Myanmar. Ethics approval was obtained from the Ethics Advisory Group, The International Union Against Tuberculosis and Lung Disease, Paris, France, and Myanmar Medical Research Centre. The need for informed patient consent was waived by the Ethics Committees as the data were obtained retrospectively from the electronic data bases in The Union and Public Health Laboratory, Mandalay. Patient records extracted from the database were anonymized and de-identified before analysis (S1 Dataset).

### Study Design

This is a retrospective cohort study involving a record review.

### Study Setting

#### General

Myanmar is situated in South East Asia and has an estimated population of 51.4 million, of whom 60% live in rural areas [7]. The country is divided administratively into 14 states/divisions, 65 districts and 325 townships. HIV prevalence in the adult population (above 15 years of age) declined from 0.94% in 2000 to an 0.5% in 2013, but higher HIV prevalence is found in key populations–female sex workers (6%), men who have sex with men (7%) and male injecting drug users (17%)[8].ART scale up continues as the key intervention for treatment and prevention of HIV, with the plan to place nearly 110,000 on ART by 2016 [5].

#### Integrated HIV Care Program

Since 2005, The Union has been implementing the ‘Integrated HIV Care program’ (IHC) in the public sector in 14 townships through 36 service delivery clinics in Myanmar. The program is supported by Total/Yadana (Oil and Gas Production) project in Myanmar (2005–2016) and the Global Fund Against AIDS, Tuberculosis and Malaria (2011–2016) where adults and children receive ART free of cost. Since 2005, the program has used d4T- and AZT-based regimens as first line treatment. When patients start ART, they have a range of laboratory tests carried out which includes full blood count, biochemistry, CD4 cell count and hepatitis B and C serology. Patients are seen every three months at which CD4 cell counts are done and other investigations according to the type of ART regimen, such as hemoglobin for patients on AZT-based ART and liver function tests for those on nevirapine.

#### Viral load screening algorithm

Patients alive on d4T-based or AZT-based ART who presented to one of the 36 clinics in the Integrated HIV Program from August 2012 onwards were offered HIV RNA viral load testing. Blood samples were taken and transported to the public health laboratory in Mandalay where viral load testing was performed. Plasma samples were tested for HIV RNA using real time PCR (RT-PCR) (GENERIC HIV Viral Load, Biocentric, Bandol, France) with a linear dynamic range of 250 to 10 million copies/ml. Those with a detectable viral load >250 copies/ml (the lower limit of detection of the viral load machine) were provided with adherence counseling within 3 months of testing and this was conducted by nurses in special counseling sessions within one of three hospitals in Mandalay. Three months later a repeat test was carried out for HIV RNA using the same methodology and patients with a detectable viral load >1000 copies/ml were considered to be first-line ART failures, in line with the WHO 2013 Consolidated ART Guidelines [3]. Patients who had failed treatment were initiated on second line ART consisting of TDF-3TC-LPV/r.

#### Viral load testing

Viral load testing was started in August 2012, but not all patients received testing for two main reasons. First, there were resource constraints with a maximum of 80 viral load tests being done in the laboratory each week and therefore some patients eligible for testing could not be tested because the testing threshold number had been reached. Second, some clinicians did not refer patients for testing based on the belief that AZT was a satisfactory ART regimen.

### Study population

Adults (15 years and above) with HIV infection retained in care for more than 12 months, who had no clinical or immunological evidence of failure on d4T-based or AZT-based ART and who underwent the viral load screening algorithm between August 2012 and March 2014 were included in the study.

### Study variables

Study variables at baseline included: age, sex, education status, alcohol consumption, whether treatment was initiated at a private ART clinic before enrolling at the Integrated HIV care clinic, ART regimen, WHO clinical stage, presence of opportunistic infection including tuberculosis, and CD4 cell count. These were collected from a patient electronic data base maintained at the Union. For viral load screening, the results of the first and second viral load measurement were documented and data obtained from the laboratory electronic data base.

### Analysis and statistics

All data were extracted into an EXCEL file. Data were imported to EpiData for analysis using EpiData Analysis software (version 2.2.2.182, EpiData Association, Odense, Denmark). Frequencies and proportions were calculated. Baseline characteristics were assessed in relation to ART failure using relative risk (RR) and 95% confidence intervals (CI) with significance being set at 5% (*P*<0.05). The variables of age group and private ART, which were significant at a *P* value of less than 0.1 in the univariate analysis, were included in the multivariate analysis. Adjusted relative risks were calculated with log binomial regression analysis using STATA version 12.1.

## Results

A total of 10456 patients were eligible for viral load testing. Of these, 5318 were not tested because of clinicians maintaining patients on AZT-based ART (N = 4403) or because of the resource constraints already described (N = 915). Of the 5138 patients who underwent the viral load testing algorithm, 204 had incomplete records and were excluded from further analysis.

There were a total of 4934 patients [median (IQR) age 36 (31–41) years] who were tested for HIV RNA viral load and these were all included in the analysis. The median (IQR) duration of ART was 28 (16–35) months. Most patients were aged 25–64 years, most had received secondary school education, and two thirds had never consumed alcohol. Nearly one quarter had been initially treated with antiretroviral drugs in the private sector before enrolling in the Integrated HIV Care Program. d4T-based ART was being taken by 82% and AZT-based ART by 18% of patients. At the time of registration for ART, 65% of patients were in WHO Clinical Stage 3 or 4 and 50% had a CD4 cell count <200 cells/uL. Just over 40% of patients had an opportunistic infection, of whom half had TB.

**Fig 1:**
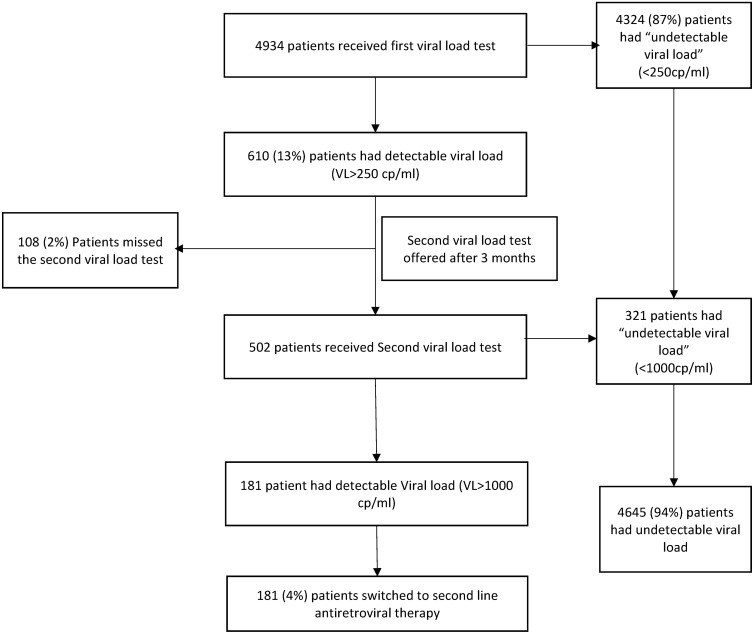
Management and results of the screening algorithm to determine antiretroviral therapy failure in HIV-infected patients, Mandalay, Myanmar.

The management and results of viral load screening are shown in Fig 1. There were 4324 (87%) who had an undetectable viral load at the first test and 610 patients with a viral load >250 copies/ml. Of these, 502 had a second viral load test at six months, of whom 181 had a viral load >1000 copies/ml, signifying ART failure. There were 108 who failed to have the second viral load test at six months. Of these, 69 patients had a viral load >1000 copies/ml on the first test and they were all placed by clinicians on second line ART. The remaining 39 patients had a viral load between 250 and 1000 copies/ml on the first test, and they were changed to TDF-based first-line ART without a second viral load test being done. Altogether, of patients being screened with the viral load testing algorithm, there were 94% with an undetectable viral load, 4% who had ART failure and 2% who did not follow the accepted viral load testing algorithm and for whom viral load status could not be properly assessed.

Baseline characteristics associated with ART failure are shown in Table 1, with those who did not complete the testing algorithm (N = 108) being excluded from the analysis. In the adjusted analysis, patients aged 15–24 years (aRR 2.4, 95% CI: 1.5–3.8) compared to 25–44 years and those who were initially treated with antiretroviral drugs in the private sector before enrolling in the Integrated HIV care program (aRR 1.6, 95% CI: 1.2–2.2) compared to the public sector had a significantly increased risk of ART failure.

**Table 1:** Association between baseline characteristics and the development of antiretroviral therapy failure in HIV-infected patients starting stavudine-based or zidovudine-based antiretroviral therapy, Mandalay, Myanmar.

Baseline characteristics	Treatment Failure N (%)	No failure N (%)	Unadjusted RR (95% CI)	P value
Total	181 (4)	4645 (96)		
**Age group in years:**				
15–24	18 (8)	206 (92)	2.3 (1.4–3.7)	<0.001
25–44	136 (4)	3753(96)	1	
45–64	26 (4)	661 (96)	1.1 (0.7–1.6)	0.7
>64	1 (<1)	21 (<1)	1.3 (0.2–8.9)	0.8
Not recorded	0	4 (<1)	-	-
**Sex:**				
Male	103 (4)	2556 (96)	1.1 (0.8–1.4)	0.6
Female	78 (4)	2087 (96)	1	
Not recorded	0	2 (<1)	-	
**Education status:** ^a^				
Educated	162 (4)	4033 (96)	1.2 (0.8–2)	0.4
Uneducated	17 (4)	524 (96)	1	
Not recorded	2 (2)	88 (98)	0.7 (0.2–3)	0.6
**Alcohol status:** ^b^				
Habitual	11 (4)	303 (96)	0.9 (0.5–1.7)	0.7
Social	34 (5)	771 (95)	1.1 (0.8–1.6)	0.6
Never	126 (4)	3190 (96)	1	
Not recorded	10 (3)	381 (97)	0.7 (0.4–1.3)	0.2
**Private ART:** ^c^				
Yes	122 (5)	3548 (95)	1.6 (1.2–2.1)	0.004
No	57 (4)	1039 (96)	1	
Not recorded	2 (4)	58 (96)	1.0 (0.3–4.0)	1
**Type of ART:** ^d^				
d4T/3TC/NVP	102 (4)	2497 (96)	1	
d4T/3TC/EFV	45 (3)	1299 (97)	0.8 (0.6–1.2)	0.3
ZDV/3TC/NVP	19 (4)	444 (96)	1.0 (0.6–1.7)	0.8
ZDV/3TC/EFV	15 (4)	405 (96)	1 (0.5–1.5)	0.7
**Opportunistic infection (OI)** ^e^				
None	106 (4)	2717 (96)	1	
Any OI except TB	36 (4)	957 (96)	1.0 (0.7–1.4)	0.9
TB (all types)	39 (4)	971(96)	1.0 (0.7–1.5)	0.9
**WHO Clinical Stage:**				
1	26 (4)	805 (96)	1	
2	32 (4)	822 (96)	1.2 (0.8–2)	0.4
3	102 (4)	2293 (96)	1.3 (0.9–2)	0.1
4	21 (3)	703 (97)	1 (0.5–1.7)	0.7
Not recorded	0	22	-	
**CD4 cell count (cells / uL):**				
0–100	52 (5)	1176 (95)	1	
101–200	48 (4)	1147 (96)	1 (0.7–1.3)	0.7
201–250	14 (5)	458 (95)	0.7 (0.4–1.2)	0.2
251 and above	34 (4)	841 (96)	1 (0.6–1.4)	0.6
Not recorded	33 (4)	1023 (96)	0.8 (0.5–1.1)	0.1

^a^ Educated = Passed the secondary school and able to write and read; No Education = under primary school level, not able to read and write

^b^ Social drinker = a person who drinks alcohol repeatedly in small quantities, Habitual = a person who drinks an excessive amount of alcohol

^c^ A patient who started antiretroviral treatment in the private sector and then moved to the integrated HIV programme

^d^ d4T = stavudine; 3TC = lamivudine; NVP = nevirapine; EFV = efavirenz; AZT = zidovudine

^e^ Any opportunistic infection that was present at the time of starting antiretroviral therapy

## Discussion

This is the first report from Myanmar of a strategy of viral load screening to assess whether patients on d4T-based or AZT-based first line ART for over 12 months and who had no clinical or immunological evidence of ART failure could safely change to a TDF-3TC-EFV first line regimen or needed switching to second line therapy. The majority of patients who were enrolled in the study (94%) completed the viral load screening algorithm and had no evidence of virological failure. A small proportion (4%) had viral load failure and needed to be switched to second line therapy, with the main risk factors being young age of 15–24 years (aRR-2.4) and previous HIV treatment in the private sector (aRR-1.6).

The strengths of this study were the large number of patients being evaluated for viral load and the implementation of this activity within the context of a national program. Results can therefore be generalized to a wider population. Baseline data collection was adequate with only a few missing results. We also used STROBE guidelines and sound ethics principles for the conduct and reporting of this observational study [9,10].

There were four main limitations. First, resource constraints meant that some eligible patients were not tested. Second, there were many patients on AZT-based ART who were not given the opportunity of viral load testing due to decisions by clinicians to maintain them on this therapy. Although we do not have precise quantification of why this was done, there are some possible reasons. There were large stocks of AZT in the pharmacies at the time and some clinicians felt these should be used before changing to TDF. The 2013 WHO Guidelines moreover emphasized the phasing out of d4T, and while TDF was recommended as the preferred first-line option, AZT was also mentioned as an alternative option [3]. Clinicians might have thought that this supported their decision to maintain AZT-based ART. Third, there was a small group of patients who did not follow the viral load testing algorithm and in whom a decision about switching to second-line therapy or changing to first-line TDF-ART was made on the basis of just one viral load test. Decisions based on one viral load test are inappropriate. It is well known that viraemia can be successfully reversed following an intensified adherence intervention in the majority of patients [11–13], and this was what was found in our study. Fourth, since HIV drug resistance was not routinely monitored in the study sites, we did not have information on HIV drug resistance patterns and also on the risk factors for the accumulation of nucleoside reverse transcriptase inhibitor (NRTI)-associated mutations.

The main risks for viral load failure were young age between 15–24 years and previous treatment in the private sector. Adolescents (defined as 10–19 years) are a vulnerable group that have been identified as being at particularly high risk of poor adherence to treatment, loss to follow-up and treatment failure [14–16] and this may be a reason for why the young patients in our study were more at risk of ART failure. Previous treatment in the private sector where patients have to pay for medication is also well recognized as a risk factor for virological failure, because costs of treatment, which may be difficult to afford, often lead to interruption of treatment or patients halving their doses of medication [17,18].

There are some important implications from this study. First, there are good reasons to change to a TDF-based regimen in line with the policy decision in Myanmar. The drug is patient friendly through its once daily dosing, there are few adverse side effects, few drug-drug interactions, the chances of accumulation of drug resistance are lower compared with other nucleoside reverse transcriptase inhibitors, and the drug has dual action against Hepatitis B [3,19,20,21]. Patients being maintained on AZT-based ART should change to TDF-based ART. Second, viral load testing, while costly, is the current recommended way to monitor response to ART as it is more sensitive and specific than using clinical assessment and/or CD4 testing [22–25]. This again supports Myanmar’s policy for this approach. Third, the strategy of testing patients on d4T-based or AZT-based therapy before they changed to TDF-based ART despite no clinical or immunological evidence of failure was sound as a small proportion of patients was found to have ART failure. It would have been bad clinical and public health practice to have started them on a first-line regimen. If ART failure is not identified early, this can lead to accumulation of drug resistance which in turn increases morbidity and mortality [26,27].

Based on this experience, we would suggest that this strategy be considered in other HIV/AIDS programmes in Myanmar and other countries, subject to sufficient human and financial resources. Finally, if this strategy is taken up it will be important to ensure adherence to protocol and prevent decisions on treatment being made based on one viral load test as happened in a few patents in our study.

In conclusion, we were able to enroll nearly 5000 patients with HIV infection and on d4T-based or AZT-based ART for at least 12 months into a strategy of viral load testing before considering a change to TDF-based ART. The large majority of these patients had no evidence of ART failure and could be safely changed to a first-line TDF-based ART regimen. However, a small proportion did have evidence of ART failure and needed to be identified and switched to second-line therapy. Little is known about the effect of HIV-1 resistance mutations present at the time of regimen switch on the response of second line therapy. A point of care genotypic resistance test coupled with VL would avoid the logistical challenges and delays associated with centralized VL testing, but this of course requires resources. This should prevent them accumulating drug resistance mutations and improve their long-term prognosis. This strategy could be considered by HIV/AIDS programmes in Myanmar and other countries.

## Supporting Information

S1 Dataset Viral load testing and treatment failure among HIV patients, Myanmar. (DTA)
